# Components of Behavioral Parent Training for Children With Attention-Deficit/Hyperactivity Disorder: A Series of Replicated Single-Case Experiments

**DOI:** 10.1177/01454455231162003

**Published:** 2023-04-13

**Authors:** Rianne Hornstra, Patrick Onghena, Barbara J. van den Hoofdakker, Lianne van der Veen-Mulders, Marjolein Luman, Anouck I. Staff, Saskia van der Oord

**Affiliations:** 1 University of Groningen, University Medical Center Groningen, The Netherlands; 2 Accare Child Study Center, Groningen, The Netherlands; 3 University of Groningen, The Netherlands; 4 KU Leuven, Belgium; 5 Vrije Universiteit Amsterdam, The Netherlands; 6 Leuven Brain Institute, Belgium

**Keywords:** single-case experimental design, attention-deficit/hyperactivity disorder, children, behavioral parent training, antecedent-based techniques, consequent-based techniques

## Abstract

Behavioral parent training (BPT) is an evidence-based treatment for children with attention-deficit/hyperactivity disorder (ADHD). Stimulus control techniques (antecedent-based techniques, e.g., clear rules, instructions) and contingency management techniques (consequent-based techniques, e.g., praise, ignore) are the most common ones that are being taught to parents in BPT. However, research into the additive effects of these techniques is scarce. In this replicated single-case experimental ABC phase design, including six children on stable medication for ADHD (8–11 years) and their parents, the added efficacy of consequent-based techniques on top of antecedent-based techniques was evaluated. After a baseline period (phase A), we randomized the commencement time of two sessions parent training in antecedent-based techniques and two sessions parent training in consequent-based techniques for each child. Children’s behaviors were assessed by daily parent ratings of selected problem behaviors and an overall behavior rating. Although visual inspection showed that behavior improved for most children in both phases, randomization tests did not demonstrate the added efficacy of the consequent-based techniques on top of the antecedent-based techniques. Limitations of the study and recommendations for future single-case experiments in this population are discussed.

## Introduction

Attention-deficit/hyperactivity disorder (ADHD) is one of the most common mental disorders in children worldwide, with an estimated prevalence of 7.2% (Russell & Gajos, 2020; Thomas et al., 2015). It is characterized by age-inappropriate inattention and/or hyperactivity and impulsivity that may interfere with, or reduce, the quality of social and academic functioning (American Psychiatric Association, 2013). Evidence-based treatments for childhood ADHD include pharmacotherapy and psychosocial interventions emphasizing behavioral management principles (NICE guidelines and Dutch guidelines; Akwa, 2019; Evans et al., 2018; National Institute for Health & Care Excellence [NICE], 2018). Behavioral parent training (BPT) is a well-established and recommended first-line psychosocial intervention for ADHD. The treatment has considerable empirical support (Schatz et al., 2020), but effect sizes are moderate at best (Dekkers et al., 2022; Dias et al., 2013; Groenman et al., 2022; Hornstra et al., 2022). Thus, studies aimed at increasing the efficacy of BPT programs are warranted. Ideally, interventions should include components with the strongest evidence for a specific target group, but research into the efficacy of specific components of BPT for ADHD is scarce. More knowledge about the efficacy of specific components of BPT for ADHD could contribute to the development, improvement, and tailoring of these interventions, with the aim to eventually increase effectiveness (Schatz et al., 2020).

Instrumental learning principles are the foundation of most BPT programs for children with ADHD, and stimulus control and contingency management techniques are the main components that are being used to influence behaviors of the child by the parents (van der Oord & Tripp, 2020). Stimulus control techniques can be used to manipulate the antecedents of behavior (antecedent-based techniques); for example, providing clear instructions and rules or restructuring the environment of the child. Contingency management techniques are used to manipulate the consequences of behavior (consequent-based techniques); for example ignoring undesired behaviors and complimenting the child if it shows desired behavior. Most programs include both antecedent-based and consequent-based techniques. After the introduction of the separate techniques, the therapist teaches the parents how to combine them (e.g., parents give clear instructions to the child to clean up toys, ignore grumbling, and provide labeled praise to the child for doing the task). Despite the theoretical foundation underlying BPT programs, there is still much unknown about the efficacy of the separate components (i.e., sets of techniques) that are being used in BPT to alter the behavior of children with ADHD. Experimental research, such as microtrials and single-case experiments, are needed to more precisely identify the efficacy of the separate components (Kazdin, 2019; Leijten et al., 2021).

The aim of the current study was to evaluate the added efficacy of two sessions of consequent-based techniques to two sessions of antecedent-based techniques with a single-case experimental design. The rationale for the low number of therapy sessions for each component was two-fold. First, meta-analytic evidence in samples of children with behavioral problems suggests that briefer, focused parenting interventions may be even more effective than longer programs with more components (Bakermans-Kranenburg et al., 2003; Leijten et al., 2022; Schleider & Weisz, 2017). Second, our research group conducted a randomized controlled microtrial in medication-naïve children with ADHD, in which we demonstrated the efficacy of two brief parenting interventions in decreasing daily rated problem behaviors, that is, two parent training sessions of antecedent-based techniques or consequent-based techniques (Hornstra et al., 2021). Antecedent-based techniques showed a decrease in problem behavior immediately after the training (*d* = 0.59), while consequent-based techniques significantly decreased problem behaviors 2 weeks after the training (*d* = 0.54). Also, antecedent-based techniques significantly improved inattention symptoms, whereas this could not be demonstrated for consequent-based techniques. Both types of techniques significantly decreased hyperactivity-impulsivity symptoms, as compared to the control condition. Although this microtrial demonstrated the efficacy of both types of techniques in isolation, it remains unclear if and to what extend one component exactly adds to the other (i.e., delivering the consequent-based techniques after the antecedent-based techniques) in terms of efficacy. It could be that the effects of antecedent- and consequent-based techniques are additive when given subsequent to each other. A nullifying effect (i.e., one component cancels out the other) could also be a possibility, although this is not to be expected as whole programs generally constitute of both components and are usually effective (Dekkers et al., 2022; Hornstra et al., 2022). More knowledge about the added value of consequent-based techniques on top of antecedent-based techniques may help to better tailor BPT in the future.

Single-case experiments are an ideal way to examine the added efficacy of specific components of an intervention, because of the high level of experimental control and the repeated measurements to determine change within the individual participants (Dallery et al., 2013). They therefore gain popularity and interest within clinical behavioral research (Wendt & Rindskopf, 2020). In single-case experimental designs, an intervention is systematically implemented or omitted across multiple phases and the dependent variable is measured repeatedly and frequently in every phase (Kazdin, 2019). Randomization of the measurement times over the different phases is of importance to control for time-effects. Without randomization it could be that an observed effect of the dependent variable might have been there without the intervention. Furthermore, randomization contributes to statistical conclusion validity through specific statistical tests (e.g., randomization tests) based on the random assignment of measurement times (Tanious & Onghena, 2019). Replication of effects over multiple participants is also an important feature of single-case experimental studies because replications can test the transfer and generalizability of the causal effects. If an intervention effect is demonstrated across multiple replicated experiments, it increases the probability that this effect is caused by the intervention, instead of external events, maturation or the mere passage of time (Michiels & Onghena, 2019).

Single-case experiments that specifically explore the additive efficacy of consequent-based techniques to antecedent-based techniques when used by parents of children with ADHD have not yet been conducted. Nevertheless, the effects of parents and teachers applying consequent-based techniques to antecedent-based techniques have been examined in another population than specifically in children with ADHD. A number of single-case studies of children referred to an university-based school psychology clinic for non-compliance (Bellipanni et al., 2013; Everett et al., 2005; Roberts et al., 2008) demonstrated that antecedent-based techniques (i.e., instructions) decreased children’s non-compliance, and the addition of consequent-based techniques (i.e., contingent praise) further decreased non-compliance. However, apart from the fact that these studies did not focus exclusively on children with ADHD, they did not use randomization tests for their single-case analyses. The added value of consequent-based techniques to medication within the classroom has been examined in single-case studies exploring effects of teacher training for children with ADHD. These studies showed that behavioral procedures such as time-out (Northup et al., 1999), the implementation of a token economy in the classroom (Hoza et al., 1992), or teacher reprimands (Abramowitz et al., 1992) appeared to work in an additive manner to medication on child compliance. However, again, these single-case experiments did not make use of randomization tests or other statistical methods to analyze the cases. Therefore, caution has to be taken when attributing observed behavior change to the implementation of the sets of techniques in these studies.

In the current study we evaluated the added efficacy of consequent-based techniques to antecedent-based techniques used by parents of children with ADHD on reducing remaining problem behaviors when being treated with medication (i.e., inattention, hyperactive-impulsive, and/or oppositional behavior), adopting a single-case experimental design. We randomized the commencement time of the antecedent-based and the consequent-based techniques. Based on previous work (Bellipanni et al., 2013; Everett et al., 2005; Roberts et al., 2008), we tentatively expected that the consequent-based techniques would have an additive effect above the antecedent-based techniques.

## Method

### Design

**Figure 1.**
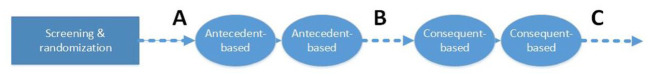
Flowchart of study procedure. *Note.* A = daily assessments in phase A; B = daily assessments in phase B; C = daily assessments in phase C.

A single-case experimental design with three phases (A-B-C) was conducted and replicated in six medicated children with ADHD. A flowchart of the study can be found in Figure 1. For each child, we randomly determined the commencement time of two antecedent-based parent training sessions and two consequent-based parent training sessions (i.e., the moment of phase change). This resulted in predetermined varying lengths of the baseline (phase A, range: 11–18 assessments), phase B (after the first and second session, range 11–18 assessments), and phase C (after the third and fourth session, range: 11–18 assessments). Outcomes were parent-rated behaviors of the child, assessed daily using ecological momentary assessment (EMA). In EMA, behaviors are repeatedly assessed in real time, in the natural environment of subjects (Russell & Gajos, 2020). With a total of 40 time points, and a minimum of 11 time points per phase, there were 36 possible assignment orders. Children were randomized to one of these possible orders at the start of the experiment using the Single-Case Randomization Tests-Package for R (Bulté & Onghena, 2013). Phase A was the baseline phase in which the daily assessments began. After that, parents received two sessions of antecedent-based techniques, and phase B started. Subsequently, parents received two sessions in which we added the consequent-based techniques, and phase C was carried out. For example, if the baseline (phase A) was 11 time points, and phase B was 11 time points, phase C was 18 time points. For an overview of the timing of the phases and sessions per case, see Appendix, Figure A. If parents canceled a session, it was rescheduled as soon as possible and the daily assessments continued until the session took place.

### Transparency and Openness

The study procedure was submitted to the Medical Ethical Committee of the University Medical Center Groningen (UMCG Research Register: 201800561), and not rated as medical scientific research. We used the Single-Case Reporting guideline In BEhavioural (SCRIBE) interventions 2016 (Tate et al., 2016) to report these single-case experiments, and we follow JARS (Kazak, 2018). The data that support the findings of this study are available from the corresponding author (RH), upon reasonable request.

### Participants and Procedure

Our sample consisted of six parents and their children with ADHD who were all being treated at an outpatient mental health clinic in the Netherlands. They were recruited between September 2018 and December 2019. Parents of children who deemed eligible received an information letter by the clinician, including the research aims and the study-procedure. If parents expressed interest in participating in the study, they received a phone call from the research team, in which more extensive explanation about the study and procedures was provided. After signing informed consent, parents and children were screened for eligibility. Inclusion criteria for the children were (a) being 4 to 12 years old; (b) having a DSM-5 based diagnosis of ADHD (confirmed with the Diagnostic Interview Schedule for Children-IV, parent interview, adapted to the DSM-5 (DISC-IV; Shaffer et al., 2000); (c) having at least six parent-rated problem behaviors to target during the sessions, derived from a comprehensive list of possible problem behaviors (Hornstra et al., 2021; Staff et al., 2021; van den Hoofdakker et al., 2007). Parents had to rate these behaviors three or higher (problems rated using a 5-point Likert scale, ranging from (1) “not severe” to (5) “extremely severe,” also see *Daily assessments*); (d) having an estimated IQ > 70 (if there was no IQ-score listed in the patient file, IQ was estimated with the subtests “Vocabulary” and “Block Design” of the WISC-III-NL or the WPPSI-III-NL); and (e) current use of psychotropic medication for ADHD (on a stable dose, according to the prescribing clinician). Exclusion criteria were (a) a clinical diagnosis of autism spectrum disorder (as reported by the parent or derived from the patient file) or (b) conduct disorder (derived from the DISC-IV [Shaffer et al., 2000], or the patient file), (c) parents received BPT in the past year, or (d) the child was not living in one household during the weekdays (as our daily assessments had to be reported by the primary caregiver; i.e., the caregiver that spent the most time with the child).

### Daily Assessments

Assessments were conducted through telephone calls (by RH) with the primary caregiver (i.e., the caregiver that spent the most time with the child), at a pre-arranged time on a daily basis (only week-days). At the start of the study, parents selected six problem behaviors they wanted to work on in the sessions. These behaviors were derived from a list of 29 possible problem behaviors including inattentive, hyperactive, and impulsive symptoms, and oppositional defiant behaviors (e.g., “disobedience,” “temper tantrums,” “not finishing tasks”; see Hornstra et al., 2021; Staff et al., 2021; van den Hoofdakker et al., 2007). Parents also had to specify in which situations these behaviors took place, situations were derived from the Home Situations Questionnaire (Barkley & Murphy, 1998). In the daily telephone calls, parents were asked if the problem behaviors had occurred that day in the specific situation. For the items scored “yes,” parents rated the severity of the behavior on a Likert scale ranging from (1) “not severe” to (5) “extremely severe.” In case the behavior in the specific situation was absent, a score of 0 was given. The selected problem behavior score was the mean score of those six behaviors. Besides that, parents also rated the overall behavior of their child on that day, on a Likert scale ranging from (1) “extremely bad” to (10) “extremely good.” The assessment thus resulted in two scores; the *overall behavior score*, and the *selected problem behavior score*.

### Intervention

Parents received four sessions of individual training, based on evidence-based programs (Barkley, 1987; McMahon & Forehand, 2003; van den Hoofdakker et al., 2007). All sessions lasted 2 hours and were delivered by the same experienced clinical psychologist/cognitive behavioral therapist (LvdVM) at a Dutch child and adolescent mental health center. In the first two sessions parents were trained in antecedent-based techniques. In the third and fourth session, parents were additionally trained in consequent-based techniques. The first two sessions were the same as the antecedent-based condition used in our previous study (Hornstra et al., 2021), the third and fourth sessions slightly differed, as the consequent-based techniques were added to the already implemented antecedent-based techniques.

The first session started with psycho-education about ADHD and executive functioning deficits. Parents learned how stimuli in situations can elicit behavior and how antecedent-based techniques can be used to support executive functioning deficits, and therefore evoke appropriate behavior and prevent unwanted behaviors to occur (Stocco & Thompson, 2015). Thereafter, based on severity, frequency, changeability, and burden to parents of that problem behavior, one of the six behaviors was selected to work on in the session, in consultation with the therapist. The therapist made a topographical analysis of the behavior and formulated a desired target behavior, together with the parents. The therapist used a functional analysis (Virués-Ortega & Haynes, 2005) to decide which antecedent-based techniques had to be part of the intervention plan. Together with the parents, an intervention plan, individually tailored to the age of the child, was designed consisting of a selection of antecedent-based techniques; that is, defining rules, giving clear instructions, anticipating misbehaviors, and providing structure in time and space. At the end of the first session, parents practiced the techniques through guided role-play or visualization. Potential barriers concerning implementation of the intervention plan at home were discussed. Parents had to implement the intervention plan immediately after the session. In the second session, last week’s intervention plan was evaluated and adapted if necessary. After that, a second behavior (from the five remaining behaviors) was selected and the same steps as in the first session were undertaken.

The third session started with psycho-education on altered reward and punishment sensitivity in ADHD. Parents learned how consequences may affect behavior, and how consequent-based techniques can be used to support motivational deficits in children with ADHD and change behaviors (Stocco & Thompson, 2015). Thereafter, a third behavior from the six behaviors was selected and an intervention plan was made, following the same process as in the other sessions. For this intervention plan, not only antecedent-based but also consequent-based techniques could be selected. Consequent-based techniques included planned ignoring, praise, rewards, and, to a lesser extent punishment. The therapist and parents made an age-appropriate plan consisting of both antecedent-based techniques and consequent-based techniques, individually tailored to the child’s age. The last session started with an evaluation of the intervention plan, and, if necessary, adaptations were made to the previous plan. A final behavior in a specific situation was selected and an intervention plan with antecedent-, and consequent-based techniques was made together with the parents.

### Fidelity

Procedural fidelity was accomplished by the use of a manual that described all components of the intervention. Adherence to the intervention protocol was measured by percentage of addressed session components. After each session, the therapist had to fill in a checklist including all session components. Additionally, all sessions were audiotaped and listened back by the first author (RH), to check if all topics were covered in the sessions, and to assess if contamination occurred. Contamination was defined as (a) consequent-based techniques that were addressed in the antecedent-based sessions, (b) questions or remarks from the therapist that could result in the parents to think of consequent-based techniques in the first two sessions, or (c) no adequate reaction from the therapist on remarks or questions from the parents that had to do with consequent-based techniques in the antecedent-based sessions, based on the procedures of H. Abikoff et al. (2013) and H. B. Abikoff et al. (2015). Adherence to the intervention protocol ranged between 96% and 100%, with no differences between the therapist-reported adherence and the audiotapes. No contamination of consequent-based techniques in the antecedent-based sessions occurred.

### Analysis

For this specific design, a proper way to estimate the number of inter-subject replications was not readily available. To estimate the planned number of single-case experiments, we used the approach for a multiple baseline design (Shadish et al., 2014). The power is a function of *n* (the number of time points per phase), *m* (the number of cases), *ρ* (the intraclass correlation), *φ* (the autocorrelation), the estimated effect size, and the set *α*. We conservatively estimated the levels of autocorrelation (*φ* = .50) and intraclass correlation (*ρ* = .50; Shadish & Sullivan, 2011). We assumed an effect size of 0.6 (based on our microtrial; Hornstra et al., 2021), and with at least 11 time points per phase, 40 time points for the comparison of the phases, and a power of 80%, six cases would be sufficient (Heyvaert et al., 2017; Horner et al., 2005; Shadish et al., 2014). As a first step, visual inspection of the data (mean and change in slope or trend; Kazdin, 2019), was used to examine the individual cases using the Single-Case Visual Analysis package of the Single-Case Data Analysis package in R (Bulté & Onghena, 2013). After that, analysis of variance *F* randomization tests were used to test whether there were any differences between the phases on either of our two outcome measures; the *overall behavior score*, and the *selected problem behavior score* for the individual cases. We expected the selected problem behavior score to decrease, and the overall behavior score to increase after implementation of the techniques learnt by parents. We calculated standardized mean differences (SMD) for the differences between the phases for each case. Additionally, we used the Single-Case Randomization Tests package (Bulté & Onghena, 2013) to analyze the effects of the antecedent-based techniques and the added effects of the consequent-based techniques. A randomization test is based on the random determination of the moment of phase change (Michiels & Onghena, 2019). We tested the null hypothesis that there was no difference between the phases A-B and B-C. The alternative hypothesis was that there was a difference in behavior between the phases A-B and B-C. The test-statistic was the absolute difference between phase A and phase B and the absolute difference between phase B and phase C. We calculated these for the overall behavior score, and the selected problem behavior score. After that, we used single-case meta-analysis to pool the *p*-values of all the cases. We used Edgington’s additive method for combining *p*-values (Edgington & Onghena, 2007), with the null hypothesis that there was no difference between the phases for any of the cases included in the experiment. In all analyses, a *p*-value of <.05 was considered statistically significant.

## Results

### Sample Characteristics

Characteristics of the children can be found in Table 1. All children were using stimulant medication for their ADHD symptoms. Four of the six children had a diagnosis of ODD, and they did not have any other known comorbid disorders (as reported by the parents and/or derived from the patient files). The total length of each experiment (first time point of phase A to last time point of phase C) differed substantially between the children. This was due to holidays and canceled sessions by parents. For an overview of the timing of the phases and sessions per case, see Appendix, Figure A. Case 1 and 6 canceled one session, case 3 and 4 canceled two sessions, case 2 canceled four sessions. All of the canceled sessions were rescheduled.

**Table 1.** Characteristics and Selected Problem Behaviors of the Six Cases.

Case	Sex	Participating parents	Age	Estimated IQ	ADHD presentation	Comorbid ODD	Selected problem behaviors (severity score at baseline)^a^	Total length of experiment	Time points per phase (A-B-C)
1	Male	Mother	11	94	Predominantly hyperactive-impulsive	Yes	1. Whining/nagging (3) 2. Demanding attention (3) 3. Temper tantrums (3) 4. Interfere when talking to others (4) 5. Dawdling (3) 6. Being overactive (5)	13 weeks	11-17-12
2	Male	Mother and father	9	93	Combined	Yes	1. Breaks stuff (3) 2. Acts like a clown (5) 3. Whining/nagging (3) 4. Being angry quickly and often (4) 5. Stealing (3) 6. Chaotic talking (5)	28 weeks	16 A-11-13
3	Male	Mother and father	11	96	Predominantly inattentive	No	1. Dawdling (3) 2. Chaotic behavior (3) 3. Being angry quickly and often (3) 4. Interfere when talking to others (3) 5. Whining/nagging (3) 6. Become unreasonably angry (3)	16 weeks	11 A-13-16
4	Female	Mother and father	10	86	Combined	Yes	1. Chaotic talking (3) 2. Dawdling (3) 3. Disobedience (3) 4. Being overactive (3) 5. Demanding attention (3) 6. Demand mothers assistance (3)	17 weeks	14 A-12-14
5	Male	Mother	10	95	Combined	Yes	1. Disobedience (3) 2. Lying (3) 3. Demanding attention (3) 4. Interrupts parents when talking to others (3) 5. Fights with sisters (3) 6. Stealing (3)	12 weeks	18 A-11-11
6	Male	Mother	8	101	Combined	No	1. Disobedience (3) 2. Fights with sister (3) 3. Does not clear away stuff (4) 4. Dawdling (3) 5. Being overactive (3) 6. Chaotic behavior (3)	14 weeks	11 A-16-13

*Note.* ADHD = attention-deficit/hyperactivity disorder; ODD = oppositional defiant disorder.

a Derived from the list of 29 possible problem behaviors (see Hornstra et al., 2021; Staff et al., 2021; van den Hoofdakker et al., 2007).

### Overall Behavior Score

**Figure 2.**
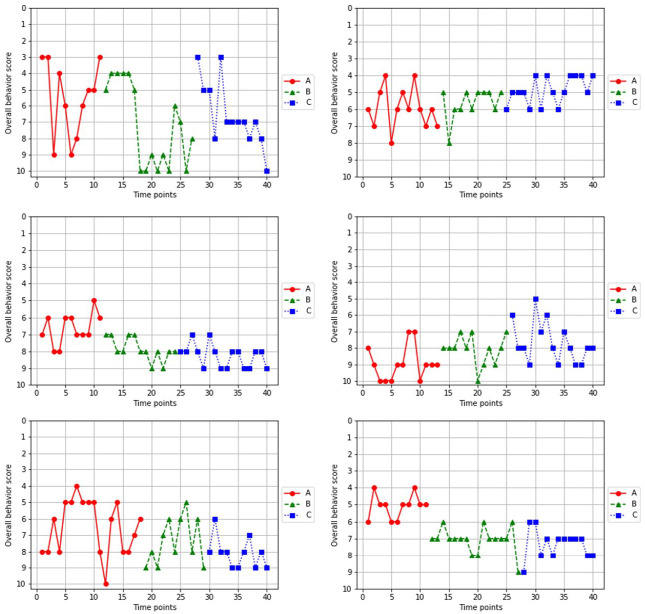
Overall behavior scores of the six single-case experiments. *Note. Y*-axis of the overall behavior scores is inverted to compare Figure 2 (overall behavior score) to Figure 3 (selected problem behavior score).

**Figure 3.**
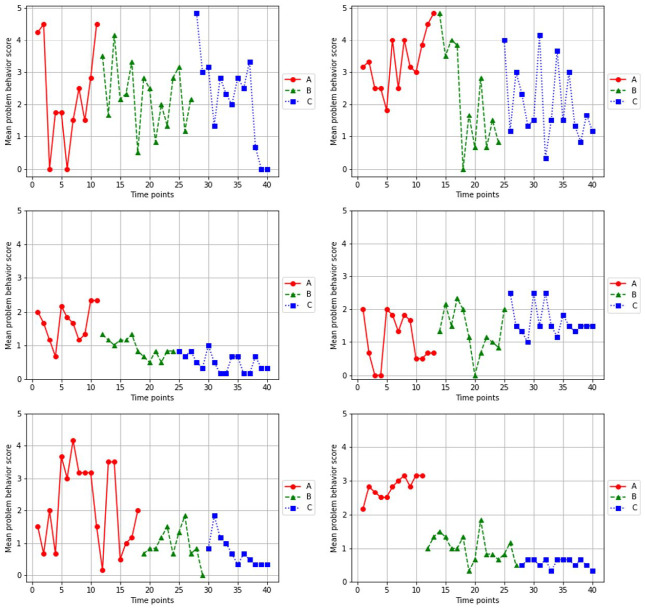
Means of the selected problem behavior scores of the six single-case experiments.

Figure 2 shows all time points regarding overall behavior scores, for all cases. Note that the *y*-axis is inverted to compare scores with Figure 3 (selected problem behavior scores). An increase in the overall behavior score resembles an improvement of the overall behavior of the children. Visual inspection of the individual cases showed that variability was high between the time points, especially for case 1. Further visual inspection tentatively indicated an improvement of the overall behavior scores between phase A and phase B for all cases, except for case 4. Between phase B and phase C, the mean overall behavior scores tentatively suggested improvement for case 2, 3, 5, and 6. For cases 1 and 4, however, overall behavior scores seemed to decrease.

The analysis of variance *F* randomization tests indicated that there were no differences between any of the three phases for each individual case (Table 2). The combined individual *p*-values of the randomization tests were not significant, indicating there was no significant difference in mean scores on the overall behavior score between phase A and phase B, and phase B and phase C.

**Table 2.** Results of the Six Single-Case Experiments.

Case	Phase A, *M* (*SD*)	Phase B, *M* (*SD*)	Phase C, *M* (*SD*)	*F*	*p*-Value	RzT, A-B	*p*-Value	SMD	RzT, B-C	*p*-Value	SMD
Overall behavior score											
1	5.55 (2.30)	7.19 (2.56)	6.54 (2.03)	*F*(2, 37) = 1.62	.86	1.64	1.00	0.72	0.65	.13	−0.25
2	4.88 (0.81)	5.64 (0.92)	5.92 (1.19)	*F*(2, 37) = 2.27	.58	0.76	.17	0.94	0.29	.33	0.31
3	6.64 (0.92)	7.85 (0.69)	8.25 (0.68)	*F*(2, 37) = 15.28	.81	1.21	1.00	1.31	0.40	.88	0.59
4	8.92 (1.04)	8.08 (0.90)	7.67 (1.23)	*F*(2, 37) = 4.81	.11	0.84	.25	−0.81	0.42	.33	−0.46
5	6.50 (1.65)	7.36 (1.43)	8.09 (0.94)	*F*(2, 22.95) = 5.34^a^	.33	0.86	.50	0.52	0.73	.73	0.51
6	5.09 (0.70)	7.06 (0.77)	7.31 (0.86)	*F*(2, 37) = 28.62	.17	1.97	.17	2.81	0.25	.75	0.32
Combi-*p*^b^							.55			.58	
Selected problem behavior score (mean)											
1	2.28 (1.62)	2.28 (1.02)	2.22 (1.40)	*F*(2, 37) = 0.01	.97	0.00	1.00	0.00	0.06	.88	−0.06
2	3.47 (0.88)	1.83 (1.37)	1.87 (1.12)	*F*(2, 37) = 4.26	.58	1.64	.17	−1.85	0.04	1.00	0.03
3	1.67 (0.54)	0.94 (0.28)	0.50 (0.27)	*F*(2, 20.66) = 24.58^a^	.78	0.73	.67	−1.36	0.44	.63	−1.53
4	1.05 (0.75)	1.58 (0.51)	1.64 (0.48)	*F*(2, 18.07) = 3.00^a^	.19	0.30	.25	0.39	0.30	.33	0.43
5	2.14 (1.28)	0.94 (0.50)	0.73 (0.47)	*F*(2, 24.62) = 8.71^a^	.72	1.20	.38	−0.93	0.21	1.00	−0.43
6	2.80 (0.32)	1.01 (0.39)	0.56 (0.13)	*F*(2, 19.42) = 226.65^a^	.06	1.79	.17	−5.54	0.45	.50	−1.14
Combi-*p*^b^							.30			.97	

*Note.* RzT = randomization test, for the randomization tests we used the absolute value; SMD = standardized mean difference.

a If variances were unequal, Welch’s *F*-test was used instead of regular *F* tests.

b Individual *p*-values were combined using Edgington’s additive approach.

### Selected Problem Behavior Score

Figure 3 displays all time points regarding means of the selected problem behavior scores, for all cases. A decrease in the selected problem behavior score resembles an improvement of behavior. Visual inspection tentatively suggested a decrease in selected problem behavior between phase A and phase B for all cases but case 4. Visual inspection of the mean of the selected problem behavior scores between phase B and phase C suggested a decrease in selected problem behavior in case 1, 3, 5, and 6. In cases 2 and 4 problem behavior seemed to increase slightly.

*F* randomization tests indicated that for each case means between any of the three phases were not significantly different (Table 2). The combined individual *p*-values of the randomization tests were not significant, indicating there were no significant differences between phase A and phase B or phase B and phase C on the selected problem behavior score.

## Discussion

This series of replicated single-case experiments was conducted to examine the added efficacy of consequent-based techniques to antecedent-based techniques on parent-reported behaviors of six medicated children with ADHD. Because consequent-based techniques were introduced subsequent to antecedent-based techniques, we can only draw conclusions about the added efficacy of the consequent-based techniques. To our knowledge, this is the first single-case experimental study that evaluated components of BPT in children with ADHD. We randomized the moment of phase change (i.e., introduction of the different types of techniques) to determine whether potential changes in behavior could be attributed to training parents in the behavioral techniques. In the current study, we could not demonstrate the added efficacy of the consequent-based techniques in decreasing selected problem behaviors and improving overall behavior of children with ADHD. Although the improvement in behavior (selected problem behaviors and overall behavior) was in the expected direction for most children, differences between the phases were not statistically significant at an individual level. When individual *p*-values were combined, we also did not find an added effect of the consequent-based techniques. Based on the current findings, we cannot make a distinction between the changes in behaviors of the children that can be attributed to the intervention, and changes that were associated with time-related confounding variables, such as history and maturation (Heyvaert & Onghena, 2014).

Despite the fact that the added efficacy of training parents in consequent-based techniques has been demonstrated in less stringent studies with non-ADHD samples or with other comparisons (Bellipanni et al., 2013; Everett et al., 2005; Roberts et al., 2008), we could not replicate this in the current series of single-case experiments. Also, we did not replicate the findings of our previous microtrial study with regard to the efficacy of the antecedent-based techniques (Hornstra et al., 2021). There are a few possible explanations for these differences in findings.

First, in contrast to the above mentioned studies, the children in the single-case experiments already used medication for ADHD, therefore possibly leaving less room for improvement. Moreover, stimulant medication could have impacted the oppositional defiant behaviors as well, as stimulant medication may significantly reduce ODD behaviors (moderate to large effects) (Pringsheim et al., 2015). However, all six children included in our study still had remaining parent selected ODD-related problem behaviors. In line with this, it should be noted that for case 4, the overall behavior score was already high (and thus not very problematic for parents) at baseline, and the selected problem behavior score was already low. Indeed, in a meta-analysis in which methylphenidate, psychosocial interventions, and combined treatments were compared, it was found that psychosocial treatment had no additional value to methylphenidate for the reduction of ADHD symptoms (van der Oord et al., 2008). Moreover, Pelham et al. (2016) examined the optimal sequencing of medication and behavioral treatment in children with ADHD. They examined whether starting treatment with either medication or behavioral treatment and, after insufficient response to the initial treatment, adding behavioral treatment or medication as a secondary step, was superior to one another. The group of children that started treatment with medication with subsequent behavioral treatment after insufficient response showed the least improvement overall. Furthermore, comparing our current findings to our previous microtrial study, starting with medication prior to the training in behavioral techniques in the present study may have negatively affected parental treatment engagement (Pelham et al., 2016), resulting in less impact of the training.

Second, behaviors selected by the parents participating in our previous microtrial (Hornstra et al., 2021) included inattentive, hyperactive-impulsive, and oppositional defiant behaviors, that were evenly distributed across participants. In the current study, most of the selected problem behaviors by parents included oppositional defiant behaviors (e.g., disobedience, being angry quickly and often, temper tantrums), and four out of six children had a comorbid diagnosis of ODD. Although representative for the problems of children with ADHD encountered in clinical practice (60% of the children with ADHD has a comorbid diagnosis of ODD; Burke et al., 2002; Connor & Doerfler, 2008), it may reflect a different sample with different needs compared to our microtrial sample. For example, particularly the affective symptoms of ODD are supposed to be associated with emotion regulation problems (Cavanagh et al., 2017). Potentially, the subgroup of children with ADHD and comorbid ODD could therefore benefit more from incorporating emotion-focused strategies in behavioral parent training (e.g., learning parents to help their children engage in problem-solving strategies and acknowledge and validate emotions) (Zachary & Jones, 2019). Future studies are needed to examine potential benefits of adding these strategies in behavioral parent training for children with ADHD and comorbid ODD.

Third, another factor that may have had an influence on the efficacy of the components is the time in between the sessions, inherent to this type of single-case experiment with randomization. Normally, as described in various BPT treatment manuals (e.g., *Parent-Child Interaction Therapy*, Ward et al., 2016; *the New Forest Parenting program*, Sonuga-Barke et al., 2006; *Incredible Years*, Webster-Stratton, 2000; *Helping the Noncompliant Child*, McMahon & Forehand, 2003; *Behavioral Parent Training Groningen*, van den Hoofdakker et al., 2007), as well as in our microtrial study (Hornstra et al., 2021), sessions are given quite intensively in a short period of time; on a regular basis in consecutive weeks. However, for the purpose of this single-case experiment the sessions had to be scheduled beforehand to ensure that there were enough time points per phase to calculate randomization tests. As a result, the period in which the four sessions were given could be long (range: 8–19 weeks). Above this, parents rescheduled, canceled, and forgot a lot of appointments, extending the period of the assessments and the study even more. This was partly a result of the design of the study, as there was a lack of continuity in appointments, but also a common pattern in BPT for parents of children with ADHD (Chacko et al., 2016). It may be that the participating parents also suffered from motivational and executive functioning difficulties and related planning problems, as ADHD is highly familial (Faraone & Larsson, 2019). The intensity of the treatment may have been insufficient, parents may have not remembered the techniques or may have forgotten to apply them. It could be that due to the longer period in which the intervention was given, parents profited less because of insufficient integration of the behavioral techniques, reduced motivation, or the recurrence of old behavioral patterns. Also the period of screening and baseline assessment (range: 21–37 days) could result in a relatively late start of the treatment. Potentially, this could have had an influence on the parents, as we know motivation is highest immediately when parents seek treatment, and waiting can result in lower intervention effectiveness (Furukawa et al., 2014; Groenman et al., 2022).

Although it was not a specific aim of this study, an interesting finding was that most children showed high variability of behavior in all phases (both in the overall behavior scores and the selected problem behavior scores), even after medication. This was not only the case in the baseline phase, but also after the introduction of the sessions in which parents were trained in the behavioral techniques. High variability in behavior may interfere with drawing clear conclusions about the effects of components (Kazdin, 2019). This variability in ADHD and related behaviors is not uncommon in children with ADHD (Schmid et al., 2020; Thunnissen et al., 2021). They often experience extremes of and shifts in positive and negative affect, resulting in a display of emotionally reactive behaviors, known as emotional dysregulation (Faraone et al., 2019; Rosen et al., 2015; Slaughter et al., 2020). Our findings emphasize the importance of an examination of behaviors with repeated measurements, instead of a single assessment to get a true picture of ADHD and related symptomatology. Single assessment ratings may be heavily influenced by current experiences and therefore biased, not accurately reflecting the actual day to day behavior of the child. Ecological momentary assessments (EMA), as used in the current study, could be an important tool to overcome these limitations, as EMA can reduce potential recall bias and improves ecological validity of findings (Russell & Gajos, 2020).

### Strengths and Limitations

This series of replicated single-case experiments provides a unique examination of the components of a BPT program for children with ADHD. To our knowledge, this is the first series of replicated single-case experiments in children with ADHD that evaluated components of BPT. However, our results have to be viewed in the light of some limitations.

First, to examine changes between different phases, single-case experiments ideally start with a relatively stable baseline phase (Dallery et al., 2013). If the dependent variable shows a stable pattern at baseline, it is easier to predict the direction of the behavior in case the intervention was not introduced, therefore increasing the change to detect potential treatment effects (Dallery & Raiff, 2014). In this study, few children showed a stable baseline phase, therefore lowering the causal inferences that can be drawn regarding the effects of the techniques. As mentioned above, in this specific ADHD population fluctuation of behaviors is more the rule than an exception. To overcome this, more time points have to be included to provide a more clear picture of behavioral patterns and to differentiate the intervention phase from the baseline phase if a true effect of the intervention is present (Krasny-Pacini & Evans, 2018). The question remains whether it is feasible to include even more assessments in ADHD populations, as parents often have similar pathology to their children, such as motivational and planning problems.

Second, the current design did not allow for the examination of the effects of the consequent-based techniques in isolation. Because the consequent-based techniques were introduced subsequent to the antecedent-based techniques, it was not possible to “unlearn” parents the use of the antecedent-based techniques. Therefore, we can only draw conclusions about the added efficacy of the consequent-based techniques.

Third, we assessed therapist fidelity but did not examine if parents implemented the techniques correctly at home. Although assessment of parental behaviors may have been difficult considering the long period in which the intervention was provided, the large time-investment and the possible Hawthorne effect (i.e., parents modify their behavior, in response to their knowing of being observed), future studies could include for example audiotaped assessments of the interaction between the parent and the child at home to examine procedural fidelity (Herbert et al., 2013).

Fourth, we did not assess whether parents fully mastered the techniques, as was done in some other behavioral parent training programs (e.g., Thomas et al., 2017). It could therefore be that parents did not fully master the techniques of one phase before they continued with the next phase, potentially influencing our results.

Fifth, although we aimed to have limited between-case variability (i.e., one therapist, same setting, all children on stimulant medication), there were some differences between the cases (e.g., one parent participating/two parents participating in the training sessions). Future single-case experiments can reduce this between-case variability, by only including families with one or two participating parents.

Lastly, it should be noted that we did not collect information about medication adherence systematically. It could be that suboptimal medication adherence was associated with less improvement or even worsening of behavior (Charach & Fernandez, 2013). Also, we did not have ethical permission to assess the patient files after the end of this series of single-case experiments, so we did not have access to information about mental health care after parents completed the study. Future studies should gather such information to follow the course of further treatment.

## Conclusion

With this single-case experiment, we aimed to examine the added effects of consequent-based techniques on top of antecedent-based techniques on behavior problems of medicated children with ADHD who showed remaining behavioral problems. Although we could not demonstrate the added efficacy of consequent-based techniques, this study provides some useful insights for future studies. Potentially, time in between sessions, and sample characteristics such as use of medication may have a negative influence on the efficacy of a training in parenting techniques. Also, the variability in behaviors of children with ADHD is high, therefore we recommend future studies to carefully consider whether to make use of a single-case experimental design and, in case this is the preferred design, to include enough cases or time points to demonstrate the efficacy of components or interventions.

## Figures and Tables

**Figure A.**
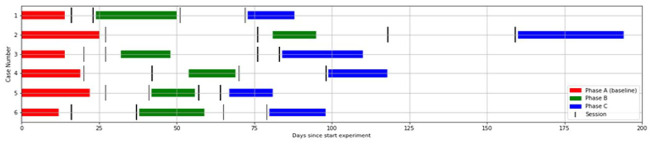
Timing of the phases and sessions per case.
